# Epigenetic Basis of Stress-Induced Central Nervous System Disorders: Therapeutic Approaches

**DOI:** 10.3390/biology15050378

**Published:** 2026-02-25

**Authors:** Yuriy Udalov, Yulia Kochenkova, Olga Kasymova, Tatiana Astrelina, Vasily Pustovoit

**Affiliations:** State Research Center—Burnasyan Federal Medical Biophysical Center of Federal Medical Biological Agency, Moscow 123098, Russia

**Keywords:** stress-induced disorders, epigenetic regulation, microRNAs, hypothalamic–pituitary–adrenal axis, BDNF, histone acetylation, post-traumatic stress disorder, depression

## Abstract

Accumulating evidence indicates that epigenetic and post-transcriptional mechanisms interact to shape stress vulnerability and the adaptive capacity of the central nervous system (CNS). This review aimed to identify molecular markers with potential prognostic value for stress-induced CNS disorders. We analyzed 93 publications (2008–2025) identified in PubMed, Scopus, Web of Science Core Collection, and the Cochrane Library, including 80 original experimental and clinical studies, as well as 13 reviews and meta-analyses addressing epigenetic regulation, hypothalamic–pituitary–adrenal (HPA) axis function, CNS remodeling, and therapeutic or environmental modulation in stress-exposed models and clinical cohorts with stress-related disorders. Across studies, altered methylation of *NR3C1*, *FKBP5*, and *BDNF*, reduced hippocampal histone acetylation, and shifts in microRNA profiles (miR-16, miR-124, miR-132, miR-135a, miR-34c) were repeatedly associated with HPA axis dysregulation, limbic system remodeling, and phenotypes relevant to PTSD and depression. Evidence further suggests that at least some of these signatures show partial reversibility, with modulation reported after pharmacological interventions (e.g., SSRIs, histone deacetylase inhibitors, FKBP51 inhibitors, ketamine) and non-pharmacological approaches (e.g., physical activity, social support) in animal models and, to a lesser extent, in clinical and observational studies. We conclude that targeted modulation of specific epigenetic and post-transcriptional pathways supports the development of candidate biomarkers and may inform stratified prevention and treatment strategies for stress-induced CNS disorders, while acknowledging that further validation in large, well-characterized cohorts is required.

## Simple Summary

Stress can lead to long-term changes in the central nervous system and contribute to disorders such as depression and post-traumatic stress disorder. This review examines how epigenetic and post-transcriptional mechanisms influence the brain’s vulnerability to stress. We analyzed studies showing that chronic stress alters methylation of key stress-response genes, histone acetylation levels, and the activity of specific microRNAs. These alterations are associated with dysfunction of the hypothalamic–pituitary–adrenal axis and structural and functional remodeling of limbic and cortical brain circuits. Importantly, many of these changes are reversible: both pharmacological interventions and non-pharmacological approaches can normalize epigenetic patterns and improve outcomes. Therefore, targeted modulation of these molecular pathways provides a promising foundation for developing candidate biomarkers and informing stratified, mechanism-based prevention and treatment strategies for stress-induced central nervous system disorders.

## 1. Introduction

Stress is a universal biological adaptation mechanism that maintains homeostasis under exposure to internal and external factors [1]. Stress arises from the interaction of internal and external stressors that challenge the homeostasis of the organism. Internal (endogenous) stressors include chronic somatic diseases, persistent pain, metabolic disturbances, and circadian rhythm disruption, whereas external (exogenous) stressors encompass psychosocial adversity, social isolation, traumatic life events, and extreme environmental conditions. Short-term stressors, defined as acute, time-limited challenges such as brief episodes of physical exertion, pain, hypoxia, or discrete psychosocial events (e.g., public speaking or examinations), activate the hypothalamic–pituitary–adrenal (HPA) axis and the sympathoadrenal system, thereby mobilizing the energetic resources of the organism while typically remaining within the adaptive (eustress) range [2]. When stressors are prolonged or recur, adaptive mechanisms progressively lose plasticity, which results in structural and functional remodeling of the central nervous system (CNS) and the development of stress-induced disorders [3]. Chronic or repeated stressors induce persistent structural and functional changes in stress-sensitive brain regions, including the hippocampus, amygdala, prefrontal cortex, and locus coeruleus. These alterations comprise dendritic retraction and reduced spine density in the hippocampus and medial prefrontal cortex, amygdala hyperactivity, disrupted cortico–limbic connectivity, and modified noradrenergic tone, which together contribute to cognitive impairment, anxiety, and affective dysregulation. These macro- and microstructural changes are described in more detail in Section 3.1.

Stress-induced central nervous system disorders encompass a spectrum of conditions in which chronic or severe stress acts as a key etiological or modifying factor. The most extensively studied entities include post-traumatic stress disorder (PTSD), major depressive disorder (MDD), generalized and social anxiety disorders, and stress-related cognitive decline, often accompanied by sleep disruption and somatic comorbidities. These clinical phenotypes share common mechanisms of HPA axis dysregulation, sympathoadrenal hyperactivity, and epigenetic remodeling of stress-related genes. At the molecular level, chronic stress is accompanied by consistent epigenetic alterations, including hypermethylation of *NR3C1*, demethylation and promoter hypermethylation of *FKBP5* in risk groups, promoter-specific hypermethylation of *BDNF*, reduced histone H3 and H4 acetylation at neuroplasticity-related loci, and dysregulation of microRNAs such as miR-16, miR-124, miR-132, miR-135a, and miR-34c [4]. These changes modify glucocorticoid receptor sensitivity, BDNF-dependent signaling, and serotonergic neurotransmission, thereby linking environmental stress exposures to long-lasting shifts in brain function and structure. Together, these epigenetic and post-transcriptional mechanisms shape stress resilience and vulnerability in stress-induced CNS disorders [5]. Although depression and anxiety represent the most intensively studied outcomes of chronic stress, epigenetic remodeling of stress-related pathways has also been linked to post-traumatic stress disorder, stress-associated cognitive decline, first-episode psychosis, and other neuropsychiatric and somatic conditions. In this review, we focus on stress-induced CNS disorders with the most consistent epigenetic and neuroimaging evidence, while acknowledging broader systemic consequences of chronic stress. In selecting the core molecular targets for this review, we focused on epigenetic loci and microRNAs that met three a priori criteria: (i) replicated associations with stress exposure or stress-related disorders in several independent human studies, (ii) convergent support from experimental animal models, and (iii) mechanistic plausibility within glucocorticoid receptor, BDNF-dependent, and serotonergic signaling pathways. At the same time, we acknowledge that the available evidence is not fully consistent across all studies, particularly with respect to the direction and magnitude of *FKBP5* methylation changes and microRNA alterations in different clinical subgroups.

Hypermethylation of the *NR3C1* promoter [6], encoding the glucocorticoid receptor, and dysregulated FKBP5 expression [7], which modulates glucocorticoid sensitivity, are associated with impaired negative feedback within the HPA axis and increased susceptibility to stress. These epigenetic changes are accompanied by reorganization of signaling pathways that control neuroendocrine homeostasis and stress resilience [2,8]. Imbalance between histone acetylation and deacetylation critically shapes the stress response, as both acute [9] and chronic stress [10] are associated with reduced H3 acetylation at promoter regions of the brain-derived neurotrophic factor gene (*BDNF*). Post-transcriptional regulators further refine the stress response: microRNAs such as miR-16, miR-124, miR-132, and miR-135a influence the expression of genes responsible for neuroplasticity and BDNF-dependent signaling [11]. The accumulated evidence supports an integrated contribution of epigenetic and post-transcriptional mechanisms to stress vulnerability and the adaptive capacity of the CNS.

Emerging therapeutic approaches aim to modulate these stress-associated molecular pathways using both pharmacological and non-pharmacological strategies. Pharmacological interventions include selective serotonin reuptake inhibitors (SSRIs), FKBP51 inhibitors, histone deacetylase (HDAC) inhibitors, and glutamatergic agents such as ketamine, which can normalize microRNA profiles, histone acetylation, and BDNF expression. Non-pharmacological interventions, notably physical activity and enhancement of social support, have also been shown to influence glucocorticoid receptor expression, BDNF levels, and stress-sensitive microRNAs, supporting their role in comprehensive prevention and rehabilitation programs.

In this context, it is essential to identify which epigenetic and post-transcriptional mechanisms possess the greatest prognostic potential for stress-induced CNS disturbances. An additional aim of this review is to highlight current uncertainties and inconsistencies in the literature, rather than to confirm a single fixed hypothesis about any one molecular pathway. The working hypothesis of this review is that epigenetic modifications of *NR3C1* and *FKBP5*, combined with alterations in the profile of stress-associated microRNAs and histone acetylation, shape a molecular pattern of stress vulnerability that reflects individual predisposition to neuropsychiatric disorders.

## 2. Materials and Methods

This review synthesizes current data on neurobiological, epigenetic, and post-transcriptional mechanisms underlying stress-induced CNS disorders. It is best characterized as a structured narrative review with elements of systematic selection. This review was conducted in accordance with PRISMA recommendations, with a predefined search strategy and explicit eligibility criteria. A structured literature search was performed in PubMed, Scopus, Web of Science Core Collection, and the Cochrane Library.

Search queries combined free-text terms and MeSH descriptors reflecting mechanisms of stress-related pathology and their pathophysiological consequences: stress response, HPA axis, sympathoadrenal system, chronic stress, epigenetic regulation, microRNA, histone acetylation, *NR3C1*, *FKBP5*, BDNF. Boolean operators AND and OR were applied to capture publications addressing both systemic and molecular aspects of chronic stress.

Backward and forward citation searching was additionally used to include studies that substantially shaped current views on the pathogenesis of stress-associated disorders. The publication window was restricted to 2008–2025, which allowed coverage of the most recent experimental and clinical evidence. Articles in English and Russian were eligible if they met criteria of scientific validity and methodological transparency.

**Inclusion criteria.** The review included (a) original experimental and clinical studies published in peer-reviewed journals and (b) systematic reviews and meta-analyses that provided critical appraisal of the evidence base. Priority was given to work focused on chronic stress and its impact on the CNS. Eligible studies examined links between activation of the HPA axis, the sympathoadrenal system, and changes in neuroplasticity, or reported epigenetic mechanisms such as methylation of *NR3C1*, *FKBP5*, and *BDNF*, histone acetylation, and regulation of microRNAs (miR-16, miR-124, miR-132, miR-135a, miR-34c).

**Exclusion criteria.** We excluded non–peer-reviewed publications, studies lacking quantitative data or using non-validated measures of neurobiological parameters, purely behavioral studies without systemic or molecular endpoints, and small studies without appropriate control groups or adequate statistical justification of results.

For experimental studies, data extraction followed a PICO-like structure, capturing the population or animal model, type of stressor or intervention, key epigenetic endpoint, and behavioral or clinical outcome. For clinical and epidemiological studies, we applied a PECO-like logic, documenting the population, exposure type and duration, comparator (when available), and epigenetic and CNS-related outcomes. Due to substantial heterogeneity in stress paradigms, tissues, and outcome measures, the review remains a structured narrative synthesis rather than a quantitative meta-analysis.

Study selection was performed independently by two reviewers, and any disagreements were resolved through discussion until consensus was reached. To minimize systematic bias, independent data verification and cross-checking of sources were applied. The process of study identification, screening, eligibility assessment, and inclusion was conducted in accordance with PRISMA principles. Records were retrieved from PubMed, Scopus, Web of Science Core Collection, and the Cochrane Library.

In total, 93 publications were included: 80 original experimental and clinical studies and 13 review articles. Original studies were classified into four main domains: (1) epigenetic and post-transcriptional regulation of the stress response and neuroplasticity; (2) neuroendocrine regulation and functioning of the HPA axis; (3) structural and functional changes in stress-sensitive CNS regions in stress-induced disorders; and (4) effects of environmental factors and therapeutic interventions on epigenetic, immune, neurotrophic, and behavioral indices of the stress response. Quantitative characteristics of the sample are summarized in Table 1.

**Conceptual analytical framework.** To ensure comparability of data and structure the results, a classification of stress reactions was employed that grades regulatory responses from adaptive to pathological activation patterns. This framework enabled a unified interpretation of findings and stratification of studies according to the level of regulation, distinguishing neuroendocrine and molecular tiers [2,12,13,14,15].

Within this scheme, eustress is defined as short-term activation of the HPA axis and sympathoadrenal system that supports adaptation without persistent molecular consequences. Distress, in contrast, reflects sustained dysregulation of stress systems, characterized by disrupted feedback, glucocorticoid resistance, and epigenetic remodeling of *NR3C1* and *FKBP5* [1,5].

Both physical (immobilization, chronic pain, hypoxia, or metabolic restriction) and psychogenic stressors (uncertainty, social isolation, or threat) were considered, which, despite differing in their triggering pathways, converge at the level of systemic effects [2,16,17,18]. Their combined impact is viewed as a key factor driving the transition from functional adaptations to stable epigenetic changes. Application of this analytical framework provided a coherent basis for organizing the results presented in subsequent sections.

## 3. Results

In the following sections, we summarize findings from experimental and clinical studies across molecular, neuroendocrine, structural–functional, and behavioral levels. Where appropriate, we refer to Table 1, Table 2 and Table 3 for semi-quantitative overviews of the number of studies per domain, predominant methods, and the direction of effects on specific markers. Analysis of 80 original studies revealed four major domains that reflect key pathogenetic mechanisms of stress-induced disturbances:

1. Epigenetic and post-transcriptional regulatory mechanisms;
2. Neuroendocrine regulation and functioning of the HPA axis;
3. Structural and functional alterations of the CNS;
4. Effects of environmental factors and therapeutic interventions on neurobiological and behavioral indices of the stress response.

The largest group comprised studies on epigenetic and post-transcriptional regulation, demonstrating the roles of *NR3C1*, *FKBP5* and *BDNF* methylation, microRNA regulation (miR-16, miR-124, miR-132, miR-135a, miR-34c), and histone acetylation changes in the development of stress-induced disorders [19,20,21,22]. Investigations focused on the HPA axis described the regulation of corticotropin-releasing hormone (CRH)-dependent pathways [23,24,25] and cortisol-related processes [6,26,27], reflecting a combined clinical and experimental evidence base.

Reports addressing structural and functional CNS changes converged on stress- related alterations in the locus coeruleus (LC) [28,29], hippocampus [30,31,32], amygdala [33,34], and prefrontal cortex [34,35,36], supported by neuroimaging and morphometric data. Studies on environmental factors and therapeutic interventions showed that social support [37,38], physical activity [39,40], and pharmacological strategies [21,41,42,43,44] can modify the methylation of stress-system genes (*NR3C1*, *FKBP5*, *BDNF*) and HPA axis activity, accompanied by shifts in anxiety-depressive symptoms, cognitive performance, and stress resilience. Summary distribution of studies across these domains is presented in Table 1.

### 3.1. Neurobiological Basis of the Stress Response

#### 3.1.1. Hypothalamic–Pituitary–Adrenal Axis

This subsection integrates predominantly clinical and neuroimaging data with supporting experimental evidence on systemic neuroendocrine regulation. The HPA axis constitutes a key component of neuroendocrine control of the stress response. Its activation is initiated by corticotropin-releasing hormone (CRH), released by neurons of the paraventricular nucleus of the hypothalamus, which stimulates adrenocorticotropic hormone (ACTH) secretion by the anterior pituitary. ACTH, in turn, regulates glucocorticoid production in the adrenal cortex, thereby supporting metabolic and energetic adaptation of the organism [45,46]. Regulation is achieved through negative feedback mediated by glucocorticoid receptors (GR) in the hippocampus and prefrontal cortex, with the amygdala contributing to modulation of the stress response [8,23,25].

Clinical observations indicate that chronic stress is associated with persistent alterations in CRH and ACTH secretion, impaired negative feedback, and enhanced HPA axis reactivity [1,6]. These changes form a neuroendocrine profile characterized by GR hyporegulation, including elevated cortisol levels and disruption of circadian rhythmicity, which is regarded as one of the pathogenetic mechanisms of stress-induced central nervous system disorders [8]. At the same time, studies of childhood trauma often report reduced basal cortisol [26] and a blunted HPA axis response to acute stress [27] rather than sustained hypercortisolemia.

#### 3.1.2. Sympathoadrenal System

The sympathoadrenal system represents the rapid component of the stress response and provides primary mobilization of energetic and behavioral resources. Activation begins in brainstem nuclei and sympathetic centers of the spinal cord, where ascending excitation is generated and propagated to hypothalamic and cortical structures. The release of noradrenaline and adrenaline from sympathetic nerve terminals and the adrenal medulla enhances cardiorespiratory responses, increases blood glucose levels, and ensures functional readiness of the organism for action.

Noradrenergic activation in the brainstem exerts a modulatory influence on limbic and prefrontal brain regions. In particular, excitation of neurons in the LC, the central noradrenergic regulator of sympathetic activation, is accompanied by changes in prefrontal and amygdala activity [47,48], creating a neural basis for regulation of attention, anxiety, and emotional evaluation of stimuli. Excessive LC activity disrupts the balance between cognitive control and emotional reactivity, a pattern observed in post-traumatic stress disorder (PTSD) [28,29].

#### 3.1.3. Role of Limbic Structures (Hippocampus, Prefrontal Cortex, Amygdala)

Limbic structures form a functional core of stress reactivity, integrating emotional appraisal, cognitive control, and endocrine regulation. The amygdala initiates threat evaluation and activation of the HPA axis, the hippocampus mediates negative feedback within the glucocorticoid loop, and the prefrontal cortex supports cognitive integration and behavioral adaptation to stressors [1,2].

Clinical studies confirm that imbalance among these structures contributes to the pathogenesis of stress-induced disorders. In patients with PTSD, reduced functional connectivity between the amygdala and ventromedial prefrontal cortex is associated with fear generalization and impaired recognition of safety cues [28]. Neuroimaging data demonstrate reduced volumes of hippocampal subfields (CA1 and adjacent regions), correlating with the duration of traumatic exposure and dysregulation of arterial blood pressure [32]. In depression, diminished inhibitory control of the medial prefrontal cortex over the amygdala contrasts with the strong inverse correlation of activity in these regions seen in healthy individuals [34], underscoring the role of cortico-limbic dysregulation in pathological stress responses.

Experimental models have clarified molecular and cellular mechanisms of dysregulation within the limbic system. Pharmacological stimulation of NMDA receptors in the basolateral (BLA) and central (CeA) amygdala reduces motivation for exploratory behavior [49]. Chronic pain similarly provokes BLA hyperactivation, leading to functional inactivation of the medial prefrontal cortex via enhanced glutamate-mediated GABAergic inhibition of pyramidal neurons and the emergence of motivational and cognitive deficits. In the CeA, a key role has been demonstrated in the comorbid development of PTSD and alcohol dependence, mediated by increased CRF and FKBP5 expression and reduced BDNF [24]. In the hippocampus, stress exposure induces retraction and simplification of dendritic architecture [50], while chronic stress disrupts Rac1-dependent plasticity [33], changes associated with cognitive decline.

The prelimbic and infralimbic regions of the medial prefrontal cortex play largely complementary roles in the stress response. The prelimbic cortex supports expression of conditioned fear and avoidance [51], while simultaneously contributing to top-down control of excessive neuroendocrine reactions to stress [52]. In contrast, the infralimbic cortex is crucial for fear suppression [53,54] and facilitates HPA axis activation during stress [55]. Chronic stress-induced disruption of plasticity and functional balance between these regions, particularly in the infralimbic cortex and its interactions with the amygdala [35], is associated with impaired extinction, dysregulated cortisol secretion, increased anxiety, and is thought to promote the development of PTSD, anxiety and depressive disorders, and pain-related syndromes [25,56].

### 3.2. Epigenetic Regulation of the Stress Response

#### 3.2.1. DNA Methylation of Stress-Response Genes

This subsection focuses on molecular evidence from human and animal studies describing DNA methylation, histone modifications, and microRNA regulation in stress-related contexts. Chronic stress is associated with persistent cognitive impairment driven by epigenetic and transcriptional dysregulation of neuroplasticity mechanisms (Table 2). Hypermethylation of *NR3C1* in the exon 1F region in adolescents correlates with depressive symptoms, anxiety, and indices of social stress [57], and similar alterations are reported in children with a history of maltreatment [20], indicating disruption of glucocorticoid feedback and increased risk of long-term cognitive-affective disturbances. Prenatal exposure to maternal depression is linked to *NR3C1* hypermethylation in newborns and an exaggerated cortisol response to stress, consistent with early reprogramming of HPA axis regulation [6]. Postmortem hippocampal samples from men with childhood trauma reveal *NR3C1* hypermethylation that is absent in individuals without traumatic history [58], and in adult men, aggressive behavior is associated with increased methylation of *NR3C1* promoter regions 1D, 1B, and 1F in peripheral blood [22].

At the same time, multiple studies demonstrate demethylation of regulatory CpG sites within intron 7 of *FKBP5*, particularly in carriers of risk alleles such as the rs1360780 T allele [7,36,59]. This pattern leads to persistently increased *FKBP5* expression [60], reduced GR function, and altered adaptation to chronic or severe stress [7,61], thereby promoting chronification of stress states and vulnerability to anxiety disorders and PTSD [62]. In contrast to stress-induced intron 7 demethylation, promoter CpG hypermethylation of *FKBP5* with reduced gene expression has been described in depression with suicidal ideation [63].

*BDNF* methylation emerges as a sensitive epigenetic indicator of maternal prenatal stress. Severe prenatal trauma in mothers is associated with site-specific increases in *BDNF* methylation in offspring, and in children of mothers with prenatal PTSD CpG methylation in *BDNF* correlates with cortisol levels, implicating the HPA axis [64,65]. Higher *BDNF* methylation is linked to more pronounced depressive and anxiety symptoms [65]. Beyond prenatal stress, childhood trauma is also associated with elevated *BDNF* methylation in peripheral blood, with the strongest associations observed in first episode psychosis [66]. In later life, a similar *BDNF* epigenetic profile is detected in patients with depression, including those with suicidal ideation, where promoter hypermethylation coincides with reduced *BDNF* mRNA expression [63]. Collectively, the *BDNF* epigenetic landscape appears highly sensitive to early stress exposures and can persist across the lifespan, being linked to affective and anxiety psychopathology at multiple developmental stages.

Animal models provide causal evidence for links between chronic stress and epigenetic regulation of key stress response and neuroplasticity genes. In wild-type mice, *Nr3c1* hypermethylation in the hippocampus and prefrontal cortex is associated with vulnerability to social stress [67]. Chronic repeated stress in the hippocampus increases methylation of regulatory regions of *Bdnf* (exon IV) and the TrkB gene, leading to reduced BDNF/TrkB expression and behavioral manifestations of heightened anxiety [68].

#### 3.2.2. Chromatin Plasticity and Histone Acetylation in Stress-Induced Neuroplasticity Changes

Chromatin plasticity is viewed as a central element of molecular adaptation to stress. Acute immobilization produces transient reductions in *Bdnf* transcript levels and histone H3 acetylation at *Bdnf* promoters [9]. When acute immobilization coincides with a critical consolidation “window” after learning, it decreases H3K14 acetylation at the *Bdnf* promoter and impairs long-term fear memory [69].

Chronic stress models show similar directional epigenetic shifts. Chronic social defeat stress (CSDS) [10], social isolation and chronic unpredictable stress [37], and chronic variable stress (CVS) [43] are all associated with reduced histone H3 acetylation in hippocampal structures. Specifically, chronic stress lowers H3K18ac [10] and H3K9ac levels [37,42,43]. Decreased H3K18ac at *Bdnf* promoters during CSDS coincides with altered dendritic morphology [10], whereas reduced H3K9ac following social isolation diminishes *Bdnf* mRNA expression and is linked to impaired long-term memory [37]. Stress-associated decreases in hippocampal H4 acetylation have also been reported, including reduced H4K12ac in CA3 and the dentate gyrus following CVS [43]. However, these findings are not reproduced across all paradigms, and in other stress models, including CSDS [10] and acute immobilization stress [69], no changes in H4 acetylation have been detected.

Increased expression and activity of histone deacetylases (HDAC1, HDAC2, HDAC5, SIRT1) in chronic stress and other depression-like models [37,42,43,44] supports the consideration of HDACs as potential therapeutic targets. Overall, reduced histone acetylation appears to represent a convergent molecular mechanism of stress-induced changes across acute and chronic stress paradigms in animals, with functional consequences largely mediated by altered *Bdnf* mRNA and protein expression.

#### 3.2.3. Post-Transcriptional Regulation of Neuroplasticity and Serotonergic Signaling by microRNAs

MicroRNAs that regulate synaptic transmission and serotonergic neurotransmission show clear clinical relevance. Reduced miR-16 levels in cerebrospinal fluid (CSF) correlate with depression severity [70]. In peripheral blood of patients with depression, decreased circulating miR-135a [71,72] and miR-16 [72] have been reported, alongside increased miR-124 and miR-132 [21,41] and higher miR-34c levels in peripheral blood cells associated with depression and cognitive impairment [73]. Plasma miR-132 positively correlates with symptom severity, whereas higher miR-34c levels are linked to more pronounced cognitive deficits [21,73]. Consistently, pharmacotherapy partially reverses these stress-associated microRNA signatures, including normalization of circulating miR-16, miR-132, and miR-124 levels [21,41]. Across independent cohorts, these alterations show moderate reproducibility, supporting the utility of circulating microRNAs as candidate biomarkers of depression and other chronic stress-related states, as well as potential targets for mechanism-based interventions.

Experimental animal models clarify the causal role of microRNAs in persistent stress-induced alterations of CNS plasticity and behavior. Hippocampal overexpression of miR-34c impairs memory, shortens dendrites, and reduces spine density, whereas miR-34c inhibition via antagomirs restores PSD-95/mTOR expression and normalizes behavioral readouts [74,75]. Chronic corticosterone administration elevates hippocampal miR-124, followed by reductions in GR/BDNF and TrkB-ERK-CREB pathway activity. Suppression of miR-124 reverses both molecular and behavioral changes [76]. Stress vulnerability has been linked to reduced miR-135a level in the medial prefrontal cortex [77] and hippocampus [71], and experimental restoration of miR-135a expression attenuates depression-like behavior [71]. Serotonergic modulation is mediated, in part, through the miR-16-SERT axis: reduced miR-16 in the CNS and CSF associates with anhedonia and increased SERT expression, whereas miR-16 upregulation in raphe nuclei mimics antidepressant effects [19,70,78,79,80,81].

#### 3.2.4. Structural–Functional Brain Changes in Chronic Stress: Role of Epigenetic Regulation

Chronic stress is accompanied by persistent morpho-functional brain changes affecting the hippocampus, prefrontal cortex, and amygdala. Reduced methylation of *FKBP5* intron 7 has been associated with decreased gray matter volume in the inferior orbitofrontal gyrus, a region involved in emotional regulation, thus linking *FKBP5* epigenetic variation to structural brain alterations that bridge genetic and environmental risk factors [36]. Decreased BDNF expression and reduced hippocampal volume are documented in patients with a first episode of depression, reflecting impaired neuroplasticity [30], while in older adults, low BDNF levels co-occur with smaller hippocampal volume and spatial memory deficits [31]. Additionally, low miR-16-2 levels have been associated with reduced insular gray matter volume on MRI and more severe depressive and anxiety symptoms [82].

**Table 2:** Epigenetic signatures of stress-induced CNS disorders: markers, alterations, associated stress conditions, and pathological states.

Marker	Epigenetic Alteration	Associated Stress Conditions	Associated Pathological States	Authors
*NR3C1*	Hypermethylation of promoters, specifically exon 1F	Social stress, childhood trauma, maternal depression	Depression, anxiety, stress vulnerability, aggression, PTSD	[6,20,22,57,58]
*FKBP5*	Intron demethylation and promoter hypermethylation	Childhood trauma	Depression, PTSD, suicidal behavior	[36,62,63]
*BDNF*	Hypermethylation	Prenatal stress, childhood trauma	Depression, suicidal behavior	[64,65,66]
miR-16	Reduced in blood and in CSF	-	Depression, reduced insular volume	[41,70,72,82]
miR-135a	Reduced in blood	-	Depression	[71,72]
miR-34c	Elevated in blood	-	Depression, cognitive impairment	[73]
miR-124	Elevated in blood	-	Depression	[21,41]
miR-132	Elevated in blood	-	Depression, anxiety	[21,41]

### 3.3. Therapeutic Modulation of Epigenetic Targets in Chronic Stress

Contemporary studies support the feasibility of targeted modulation of stress-associated molecular cascades, including microRNAs, glucocorticoid receptor (GR), FKBP5, BDNF, and histone acetylation, which are considered at least partially reversible under chronic stress. In this section, we summarize pharmacological and non-pharmacological interventions that modulate these pathways, highlighting their primary molecular targets and associated behavioral or clinical effects. The main interventions and outcomes are listed in Table 3 [41,83].

#### 3.3.1. Pharmacological Modulation of Stress-Related Pathways

Experimental data indicate that pharmacological manipulation of GR signaling regulators can normalize both molecular and behavioral manifestations of chronic stress. FKBP5-dependent pathways are viewed as promising targets for treatment or stratification of patients with depression [36]. The selective FKBP51 inhibitor SAFit2 reduces anxiety-like behavior and prevents social avoidance in animals exposed to chronic stress without altering corticosterone levels [83].

Selective serotonin reuptake inhibitors (SSRIs) exert epigenetic actions: sertraline increases miR-16 and GR and BDNF levels while reducing miR-124 and miR-132 [40]. Citalopram similarly elevates miR-16 [40] and tends to decrease miR-132, whereas for miR-124 some studies report additional increases during treatment [20]. Such effects point to a dual nature of SSRI action, combining neurotransmitter regulation with epigenetic stabilization of transcriptional programs that support neuroplasticity.

Reduced histone acetylation at *Bdnf* promoters [9,10,43,69] and increased hippocampal HDACs expression [37,43] have been consistently associated with diverse stress paradigms, making HDAC inhibition a promising therapeutic direction. Pharmacological HDAC inhibitors normalize stress-related epigenetic alterations (Table 3), including histone acetylation [42,43] and BDNF expression [84], accompanied by antidepressant and anxiolytic effects. Furthermore, in an organophosphate-induced depression model, ketamine, a glutamatergic antidepressant, reduces HDAC expression and restores H3K9ac at the hippocampal Bdnf promoter, together with normalization of BDNF levels and dendritic architecture to values comparable with controls [44].

#### 3.3.2. Non-Pharmacological Modulation

Physical activity represents a major non-pharmacological factor capable of reshaping the epigenetic profile of stress-sensitive genes. Experimental data demonstrate that exercise in mice increases GR expression [39,40], including *Nr3c1* exon 1F, and decreases hippocampal miR-124 [40], changes associated with improved cognition and more efficient HPA axis regulation. Physical training also enhances BDNF expression in both humans and animals [85], with proposed mechanisms including reduced CpG methylation in *Bdnf* promoters [86,87], increased histone acetylation [88], and modulation of microRNA expression [89].

Social isolation and loneliness across populations are associated with epigenetic changes [90,91], which are regarded as mechanisms of stress vulnerability, such as hypermethylation of *NR3C1* and *BDNF* [38]. Conversely, supportive social environments exert a protective effect against stress-induced alterations: group housing in rodents attenuates the impact of chronic unpredictable stress on HPA parameters and hippocampal epigenetic markers compared with isolated animals [37], while in humans, broader and more supportive social networks are linked to slightly lower epigenetic age [91].

#### 3.3.3. Translational Aspects and Perspectives

Combining pharmacological and behavioral interventions positions epigenetic modulation as a key element of preventive and rehabilitative strategies in stress-induced disorders. However, translation of experimental findings into clinical practice requires clarification of dose–response relationships, temporal stability of epigenetic modifications, and duration of clinical remission [5]. Integration of experimental and clinical evidence (Table 3) suggests that targeted regulation of FKBP5, GR, and BDNF-dependent cascades can redirect the stress response trajectory from pathological toward adaptive patterns, with epigenetic mechanisms emerging as promising targets for prevention and treatment of stress-related cognitive impairments.

**Table 3:** Pharmacological and non-pharmacological modulation of stress-associated molecular markers and related behavioral or clinical outcomes.

Marker	Modulation	Mechanistic and Phenotypic Effects	Authors
miR-132	Sertraline	Decreases miR-132 levels	[41]
	Citalopram	Decreases or shows no change in miR-132 levels	[21,41]
miR-124	Sertraline	Decreases miR-124 levels	[41]
	Citalopram	Increases or shows no change in miR-124 levels	[21,41]
	Antagomir	Reduces depression-like behavior	[76]
miR-16	Sertraline	Increases miR-16 levels	[41]
	Citalopram	Increases miR-16 levels	[41]
	Antagomir	Antidepressant effect (infusion into locus coeruleus)	[78]
		Induces depression-like behavior (intracerebroventricular injection)	[70]
miR-34c	Antagomir	Decreases miR-34c levels, restores spatial memory	[75]
miR-135a	MicroRNA mimic	Increases miR-135a levels in the hippocampus, reduces depression-like behavior	[71]
	Inhibition	Increases SERT expression	[92]
BDNF	Sertraline	Increases BDNF levels	[41]
	Citalopram	No change in BDNF levels	[41]
GR	Sertraline	Increases GR levels	[41]
	Citalopram	Increases GR levels	[41]
FKBP5	Inhibition	Reduces anxiety-like behavior; prevents stress-induced social isolation	[83]
HDACs	Sodium butyrate	Increases H4K12 acetylation at hippocampal Bdnf promoters, restores BDNF expression, and is associated with antidepressant-like effects in chronic stress models	[43,84]
	Sirtinol	Increases H4K12ac/H3K9ac	[43]
	Suberoylanilide hydroxamic acid (SAHA)	Increases H3K9ac; decreases HDAC2; reduces depression-like behavior	[42]
	Ketamine	Increases H3K9ac; decreases HDAC1, HDAC3, HDAC5 levels; increases BDNF levels	[44]

## 4. Discussion and Future Directions

Neuroendocrine-epigenetic coupling in chronic stress

The combined experimental and clinical evidence indicates that HPA axis dysregulation is an early and stable marker of transition from an adaptive to a pathological stress response. Prolonged hyperactivity of the sympathoadrenal system and its central components forms the basis for chronically increased autonomic reactivity. Sustained excitation of the LC and its projections to the prefrontal cortex and amygdala promotes emotional lability, anxiety states, and elevated cardiovascular risk under chronic stress [93].

Chronic stress is further characterized by amygdala hyperactivity, reduced volume and functional activity of the hippocampus, and weakened regulatory control of the prefrontal cortex. Disrupted coordination among these structures provides a neurobiological substrate for the emotional and cognitive disturbances typical of stress-induced disorders [1,5,34]. Clinical and neuroimaging data also demonstrate convergence of epigenetic markers along the NR3C1-FKBP5 axis, microRNA profiles, and neurotrophic indices with morphometric vulnerability signatures in the hippocampus and insular cortex. This pattern supports a model in which chronic stress drives long-term structural and functional change through epigenetic remodeling of glucocorticoid and neurotrophic signaling [21,30,31,57,58,63,82].

Overall, the data confirm that stress-induced CNS disorders arise from persistent alterations in neuroendocrine and epigenetic regulation. Chronic activation of the HPA axis and sympathoadrenal system modifies GR sensitivity and reshapes methylation of *NR3C1* [20,58], *FKBP5* [7,61,62], and *BDNF* [64,65,66], together with remodeling of microRNA profiles [71,76,77,79,80] and histone acetylation changes [10,37,43] that control BDNF and other neuroplasticity-related proteins [1,3,5]. Importantly, many of these epigenetic processes are reversible, opening opportunities for targeted therapeutic modulation of the stress response at the molecular level.

Molecular and epigenetic therapeutic targets

One of the most promising directions involves modulation of FKBP5, a negative regulator of GR activity. Use of selective FKBP51 inhibitors in chronic stress models reduces anxiety-like behavior and prevents disturbances of social interaction [83]. These data indicate that targeting post-receptor stress-response cascades can help maintain balanced neuroendocrine regulation without directly altering systemic hormone levels.

Mechanisms of epigenetic regulation of neuroplasticity also represent important therapeutic targets. Increased histone acetylation (H3K9, H3K18), achieved using HDAC inhibitors or certain antidepressants, is associated with restoration of BDNF expression and normalization of synaptic plasticity and cognitive performance [42,43,44,84]. MicroRNA modulation is another promising avenue, given the consistent dysregulation of specific microRNAs in depression. Clinical data show that antidepressants such as sertraline and citalopram decrease miR-132 and miR-124 and increase miR-16, changes that parallel reductions in depressive symptomatology [21,41]. Experimental manipulation of miR-135a and miR-34c has additionally been shown to improve indices of synaptic structure and cognitive function [74,75].

Pharmacological and non-pharmacological strategies

Current pharmacological correction of stress-related abnormalities includes agents that target serotonergic transmission, glucocorticoid receptors, and epigenetic enzymes. Development of combined approaches that simultaneously influence serotonergic and epigenetic regulation is a particularly promising direction, since such synergy can restore both neurotransmitter and transcriptional control of the stress response [21,41].

Accumulating evidence indicates that non-pharmacological interventions also engage key epigenetic nodes in the pathogenesis of stress-induced disorders and may therefore enhance stress resilience. In animal models, physical activity is associated with normalization of stress-response regulation at the level of GR, BDNF, and stress-sensitive microRNAs [40], in parallel with improved cognition and more efficient HPA axis control. In the social domain, isolation and loneliness emerge as factors that maintain epigenetic vulnerability, including *NR3C1* and *BDNF* hypermethylation [38], whereas supportive environments show protective associations in both animal experiments [37] and human cohorts [91]. Together, these findings justify incorporating structured physical activity and enhancement of social support into prevention and rehabilitation programs as strategies capable of modifying epigenetic markers of stress-induced dysregulation.

Prospects for translational research

A key objective for future work is to move from descriptive models toward functional patient stratification based on epigenetic profiles. Such an approach would allow prediction of treatment efficacy and selection of individualized correction strategies for stress-induced disorders. This task requires integration of multi-omics methods (DNA methylation, microRNA expression, proteomics) with neuroimaging and detailed clinical phenotyping [5].

In the longer term, development of personalized stress-response modulation schemes that combine pharmacological and behavioral interventions may underpin preventive strategies for anxiety, depressive, and cognitive disorders. The potential of these approaches is determined by their capacity to restore epigenetic balance and neuroplasticity without imposing a continuous high pharmacological burden, thereby aligning mechanistic precision with long-term tolerability.

An important limitation of our work is its hybrid design between systematic and narrative review. Although we followed PRISMA principles, applied predefined inclusion and exclusion criteria, and summarized data in a structured way, we did not perform formal risk-of-bias assessments, meta-analytic pooling of effect sizes, or protocol registration. Together with pronounced methodological heterogeneity in stress models, populations, tissues, and analytical methods, this constrains the generalizability of our conclusions and precludes robust effect-size synthesis.

## 5. Conclusions

Integrated analysis of clinical and experimental evidence suggests that chronic stress leads to a systemic dysregulation of CNS regulatory mechanisms. Key pathogenetic components include HPA axis dysregulation, sympathoadrenal hyperactivity, and epigenetic remodeling of stress-related genes such as *NR3C1* and *FKBP5*, along with histone modifications and microRNA shifts. These changes are associated with reduced neuroplasticity, cognitive impairment, and persistent affective/emotional disturbances.

The identified links between *NR3C1* and *FKBP5* methylation, alterations in BDNF levels, histone acetylation status, and microRNA profiles (miR-16, miR-124, miR-135a, miR-132, miR-34c) support their consideration as candidate biomarkers of stress-induced pathology that require further validation in large, well-characterized cohorts. Consistency across molecular findings, neuroimaging results, and morpho-functional measures strengthens the concept of epigenetically mediated neurodysregulation in chronic stress. Further research should refine pathogenetic links between epigenetic modifications and clinical manifestations of stress-related disorders and evaluate the efficacy of targeted therapeutic interventions. Particular interest lies in epigenetic and microRNA-oriented modulation, structured physical activity, and pharmacological agents that normalize GR-, BDNF- and FKBP5-dependent pathways. Combining molecular, neuroimaging, and clinical approaches will enable development of personalized strategies for prevention and treatment of stress-induced disorders of the CNS.

## Figures and Tables

**Table 1:** Quantitative distribution of sources by thematic areas, research type, and publication years.

Thematic Area	Number of Publications (*n*)	Predominant Research Type	Publication Years	Predominant Methods
Epigenetics and microRNAs	32	Experimental (70%), Clinical (30%)	2008–2025	DNA methylation, microRNA expression analysis, qPCR
Hypothalamic-Pituitary- Adrenal Axis	9	Experimental (67%), Clinical (33%)	2009–2025	Cortisol measurement, CRH test, immunoassay
Central Nervous System and Neuroplasticity	17	Experimental, Clinical (≈50% each)	2010–2023	Neuroimaging, neuromorphological analysis
Environmental Factors and Therapeutic Interventions	22	Experimental (40%), Clinical (60%)	2011–2025	RT-PCR, Western blot, ELISA, behavioral tests

Note: CRH—corticotropin-releasing hormone.

## Data Availability

No new data were created or analyzed in this study. Data sharing is not applicable to this article.
